# Test of Strength: Figure-of-Eight versus Spiral Wrapping Technique for Fiberglass Casts

**DOI:** 10.7759/cureus.7843

**Published:** 2020-04-26

**Authors:** Blake K Montgomery, Hunter W Storaci, Nicole A Segovia, Jeffrey Young

**Affiliations:** 1 Orthopaedic Surgery, Stanford University, Palo Alto, USA; 2 Orthopaedic Surgery, Lucile Packard Children's Hospital/Stanford University, Palo Alto, USA

**Keywords:** strength, cast, fiberglass, spiral, figure-of-eight

## Abstract

Pediatric fractures are a common injury, and treatment often includes cast immobilization. For pediatric patients being treated in a cast, cast damage is among the most common reasons patients return to the emergency room. The figure-of-eight wrapping technique interdigitates layers of fiberglass which may create a stronger cast. The aim of this study was to assess the strength of the figure-of-eight wrapping technique in comparison to the spiral wrapping technique. A total of 10 casts were wrapped with a three-inch fiberglass using the spiral technique and 10 casts were wrapped using the figure-of-eight technique. Each cast was then subjected to a three-point bending test and loaded until failure using an Instron machine. The figure-of-eight technique had an average load to failure of 278.2 + 27.6 N/mm which was similar to the spiral technique’s load to failure of 281.2 + 25.4 N/mm (p=0.795). Prior to normalizing for thickness, the load to failure of the figure-of-eight technique was 949.8 + 109.5 N, which was significantly higher than the spiral technique of 868.2 + 65.1 N (p=0.038). The figure-of-eight casts were slightly thicker than the spiral casts (average 0.3 mm, p=0.004). This suggests that the thickness of the fiberglass cast may improve the strength. The figure-of-eight wrapping technique had similar biomechanical characteristics to spiral wrapping techniques. Providers should wrap in whichever technique they feel most comfortable performing as there is no difference in strength of the cast. If a stronger cast is desired, then thickness of the cast can be increased.

## Introduction

Pediatric fractures are a common injury with an annual occurrence rate of 9.47 per every 1,000 children [1]. Many pediatric fractures are treated with cast immobilization. Pediatric patients remain very active in their casts, which can ultimately lead to cast breakdown. For pediatric patients being treated in a cast, cast damage is among the most common reasons patients return to the emergency room [2].

Healthcare providers have gravitated toward stronger immobilization to withstand the day-to-day stress a cast endures. Plaster of Paris was once the primary material of casts; however, fiberglass is now the most common material used for casting, which is largely due to increased strength [3-6]. Historically, wrapping a cast with a figure-of-eight technique has been considered to provide more strength to the cast; however, there are not any biomechanical studies to support this theory. The aim of this study was to assess the strength of the figure-of-eight wrapping technique in comparison to the spiral wrapping technique.

## Materials and methods

**Figure 1:**
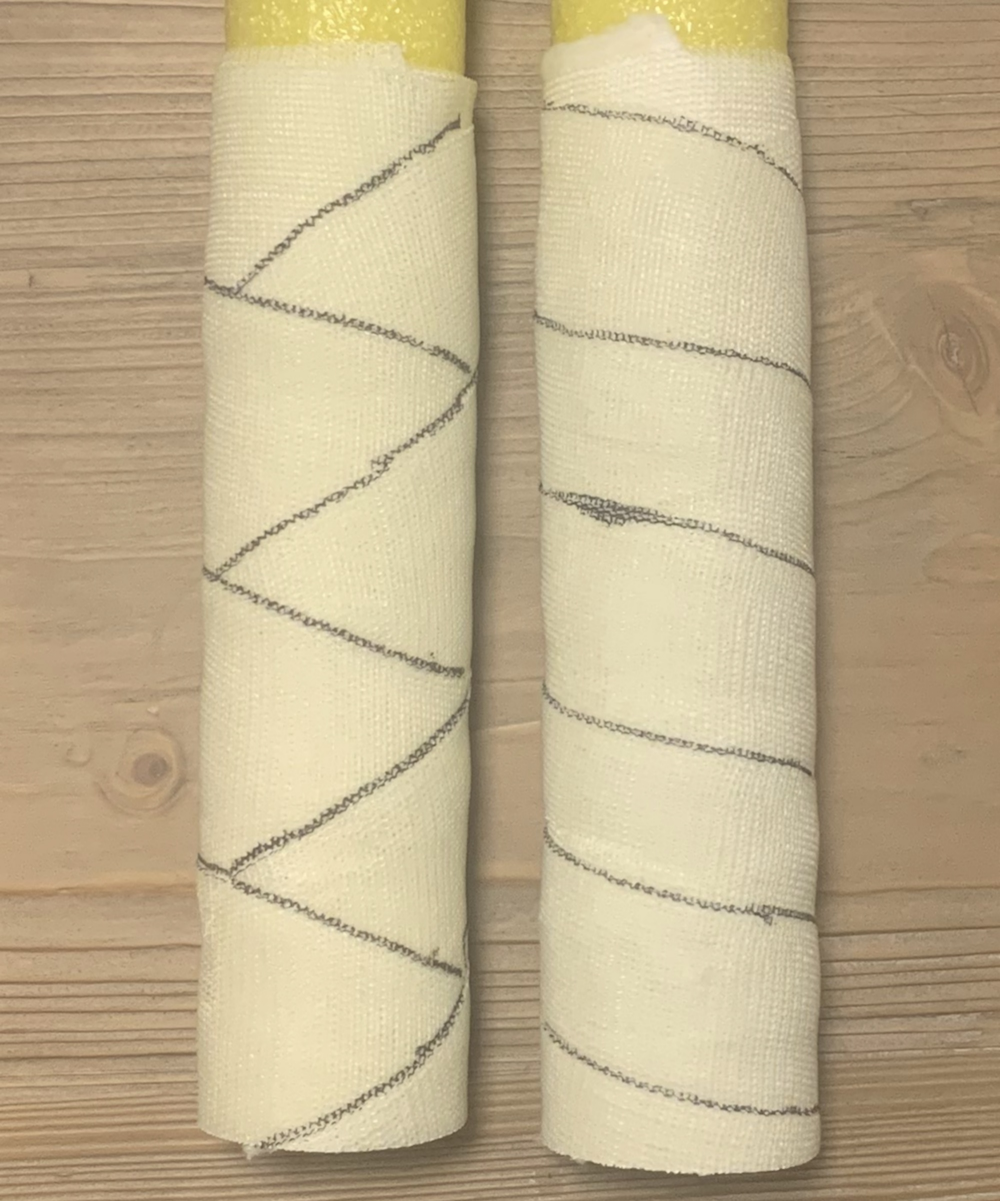
Example of figure-of-eight wrapped cast (left) and spiral wrapped cast (right).

A total of 20 polyethylene foam core cylinders were used as cast models (SR20C-Gladon Co, Oak Creek, WI). Each cast model was wrapped in one layer of three-inch cast padding with 50% overlap. A total of 10 casts were wrapped with a three-inch fiberglass using the spiral technique and 10 casts were wrapped using the figure-of-eight technique (Figure 1).

Two applications of fiberglass were used for each cast, resulting in approximately four layers of fiberglass. Each cast was applied by the same experienced orthopedic surgeon. Casts were then dried for at least two days.

**Figure 2:**
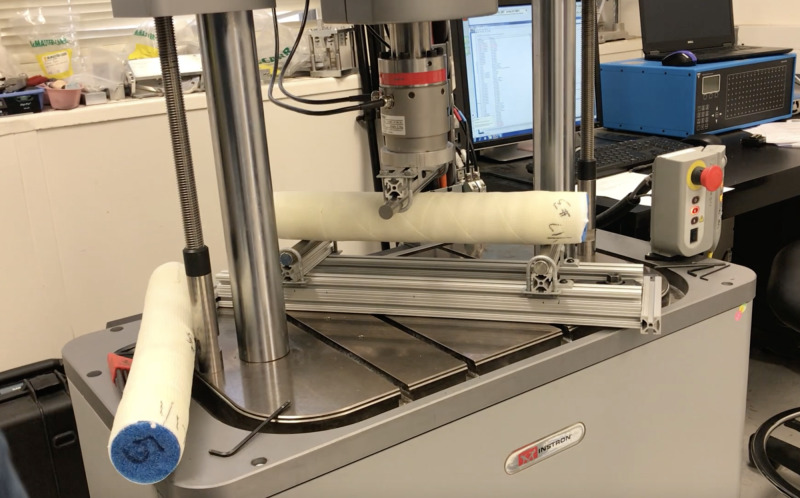
Instron three-point bending apparatus.

The three-point bending test is the most clinically relevant way to assess cast strength [7,8]. Each cast was then subjected to a three-point bending test and loaded until failure using an Instron machine, and the biomechanical properties were recorded (Figure 2).

Each cast was then split and the thickness of each cast was measured to allow for normalization. Statistical analysis was conducted with Student’s t-test with significance set to α < .05. Statistical analyses were conducted using t-tests with significance set to α < .05. Based on a power analysis, a sample size of 18 (9/group) provides at least 80% power to detect a difference of 15% in cast strength characteristics between the figure-of-eight and spiral wrapping techniques.

## Results

**Figure 3:**
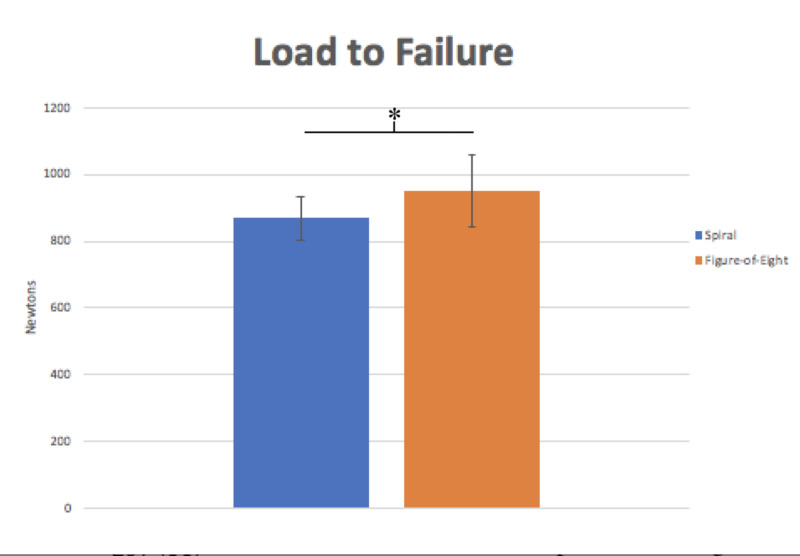
Graph displaying the load to failure of the spiral wrap versus the figure-of-eight wrap. Without normalizing for the minute thickness difference the figure-of-eight wrap appeared stronger than the spiral wrap technique. The error bars represent standard deviation. *=p<0.05

**Figure 4:**
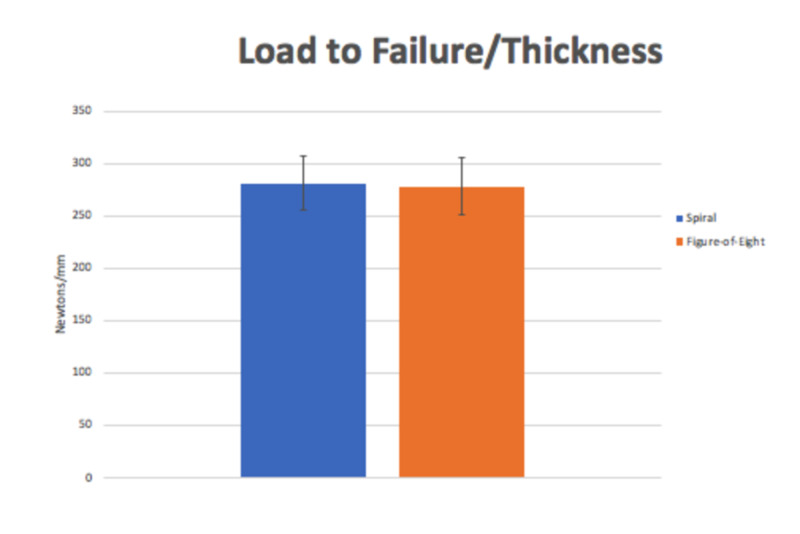
Graph displaying the load to failure of both groups after normalizing to thickness. The apparent strength difference between the group diminishes, suggesting cast thickness is the greater determinant of cast strength. The error bars represent standard deviation. *=p<0.05

The figure-of-eight wrapping technique conveys similar strength to the spiral wrapping technique. The figure-of-eight technique had an average load to failure of 278.2 + 27.6 N/mm, which was similar to the spiral technique’s load to failure of 281.2 + 25.4 N/mm (p=0.795). The stiffness of the figure-of-eight technique was 180.3 + 21.8 N/mm, which was also similar to the spiral technique’s stiffness of 186.8 + 8.5 (p=0.433) (Figures 3, 4).

Cast thickness significantly affects cast strength. The average thickness of the figure-of-eight cast was 3.4 + 0.3 mm, which was minimally, but significantly thicker than the average thickness of the spiral cast, which was 3.1 + 0.2 mm (p=0.004). Although this was a very small difference, it had profound influence on cast strength. Prior to controlling for thickness, the load to failure of the figure-of-eight technique was 949.8 + 109.5 N, which was significantly higher than the spiral technique of 868.2 + 65.1 N (p=0.038). This suggests that the thickness of the fiberglass cast has a considerable effect on the strength. 

## Discussion

Fiberglass cast application and maintenance are instrumental in the treatment pediatric orthopedic musculoskeletal ailments. Cast breakdown is problematic and often necessitates a trip to the clinic or emergency department for cast reapplication [2]. A stronger cast may prevent cast breakdown, which would decrease emergency room and clinic visits, thus decreasing healthcare costs.

Fiberglass has many favorable biomechanical properties including its strength, weight, and radiolucency [9-14]. A biomechanical study examined the properties of fiberglass cast material in comparison to plaster of Paris cast material [11]. They determined fiberglass load to failure was approximately 180 N while plaster of Paris was drastically weaker with load to failure approximately 20 N per layer of cast material. The same study also assessed the radiolucent properties of fiberglass in comparison to plaster of Paris and determined that fiberglass was more radiolucent, absorbing only half as much radiation as plaster of Paris. An additional study determined that fiberglass casts were approximately 40% lighter than plaster of Paris casts [10]. Another study of military troops that were casted with either fiberglass or plaster of Paris material showed that patients in the fiberglass group were more comfortable, able to better achieve activities-of-daily living, and felt the cast was lighter. The cost was also similar between the two groups [9]. 

New techniques have been developed that combine plaster and fiberglass (hybrid cast) in efforts to harness the best properties of both materials [10,15]. This cast provides a stronger and lighter cast than a pure plaster of Paris cast [10]. The plaster layer is applied first and allows for more molding in comparison to fiberglass [10,16]. Hybrid casts are used today in special clinical situations, but are not used routinely.

Additional interest has focused on techniques to improve strength of fiberglass casts [17,18]. One study compared six different synthetic cast materials from multiple different companies at different time points and found various differences in strength amongst the groups [17]. Another study assessed the strength of two-, three-, four-, and five-inch width fiberglass [18]. They found that five-inch width fiberglass produced the strongest cast. However, the study did not assess for casts thickness or normalize between the groups which ultimately questions the validity of the study.

The figure-of-eight wrapping technique is popular amongst cast technicians and teaching hospitals. The figure-of-eight wrapping technique consists of interdigitating the fiberglass at approximately 45 degree angles. This method is time consuming and tedious. Historically, this technique was assumed to provide additional strength to the cast construct; however, to the best of our knowledge, there are not any biomechanical assessments comparing the figure-of-eight technique to the spiral technique.

This study compared the biomechanical properties of the figure-of-eight wrapping technique to the spiral wrapping technique. Our results demonstrate that both techniques provide similar load to failure and stiffness. Interestingly, the figure-of-eight casts were slightly thicker than the spiral wrapping group (0.3 mm). This small difference in thickness attributed to approximately 9% increase in cast strength. 

The primary limitation of this study is that it is a biomechanical study, and its translation to clinical practice is not guaranteed.

## Conclusions

While fiberglass casts are currently the gold standard and are stronger than many other cast material, they still fail. Increasing cast thickness improves strength; however, figure-of-eight and spiral wrapping techniques convey similar biomechanical properties. Further research is needed to improve cast strength and prevent early cast breakdown. 
